# Abdominal wall inflammatory myofibroblastic tumor initially misdiagnosed as leiomyoma: a rare case report

**DOI:** 10.1093/jscr/rjag065

**Published:** 2026-02-12

**Authors:** Youssef T Youssef, Mohamed Baklola, Mohamed Abdelhai Mahmoud, Mohammed R Arrabyee, Eslam Ashraf Mustafa Ibrahim, Tamer Youssef

**Affiliations:** Faculty of Medicine, Mansoura University, 60 El-Gomhoria Street, Mansoura 35516, Dakahlia, Egypt; Faculty of Medicine, Mansoura University, 60 El-Gomhoria Street, Mansoura 35516, Dakahlia, Egypt; General and Endocrine surgery, Mansoura University Hospitals, 60 El-Gomhoria Street, Mansoura 35516, Dakahlia, Egypt; Faculty of Medicine and Health Sciences, Sana’a University, Wadi Dhaher Road, Sana’a, Republic of Yemen; General and Endocrine surgery, Mansoura University Hospitals, 60 El-Gomhoria Street, Mansoura 35516, Dakahlia, Egypt; General and Endocrine surgery, Mansoura University Hospitals, 60 El-Gomhoria Street, Mansoura 35516, Dakahlia, Egypt

**Keywords:** inflammatory myofibroblastic tumor, abdominal wall mass, spindle cell tumor, leiomyoma, surgical excision

## Abstract

Inflammatory myofibroblastic tumor is a rare mesenchymal neoplasm that most commonly arises in the lung and intra-abdominal soft tissues. Involvement of the anterior abdominal wall is exceptionally uncommon and can lead to diagnostic difficulty. We report a case of a 34-year-old male who presented with a painless, enlarging supraumbilical mass. Imaging demonstrated a heterogeneously enhancing lesion with intra-abdominal extension and close proximity to bowel loops. Core needle biopsy suggested a benign spindle cell tumor consistent with leiomyoma. Due to diagnostic uncertainty, complete surgical excision was performed. Final histopathology confirmed inflammatory myofibroblastic tumor with clear margins. The postoperative course was uneventful, and follow-up was arranged because of the risk of recurrence. This case highlights the diagnostic challenges of abdominal wall inflammatory myofibroblastic tumors and emphasizes the role of surgery in achieving definitive diagnosis and treatment.

## Introduction

Inflammatory myofibroblastic tumor is an uncommon spindle cell neoplasm characterized by myofibroblastic proliferation with associated chronic inflammatory infiltrates [1]. Although originally described in the lung, it has been reported in various extrapulmonary sites, including the mesentery and retroperitoneum [1]. Primary involvement of the anterior abdominal wall is rare and often mimics other benign or malignant soft tissue tumors [2]. Clinical presentation and imaging findings are frequently nonspecific, making preoperative diagnosis difficult [3]. Surgical excision remains the mainstay of management and is often required to establish a definitive diagnosis [2]. We present a rare case of abdominal wall inflammatory myofibroblastic tumor initially misdiagnosed as leiomyoma on biopsy.

## Case presentation

A 34-year-old male with a history of smoking and no prior surgery presented with a painless, progressively enlarging supraumbilical abdominal wall mass of two months’ duration. He reported no systemic or gastrointestinal symptoms, and physical examination showed no signs of inflammation.

**Figure 1:**
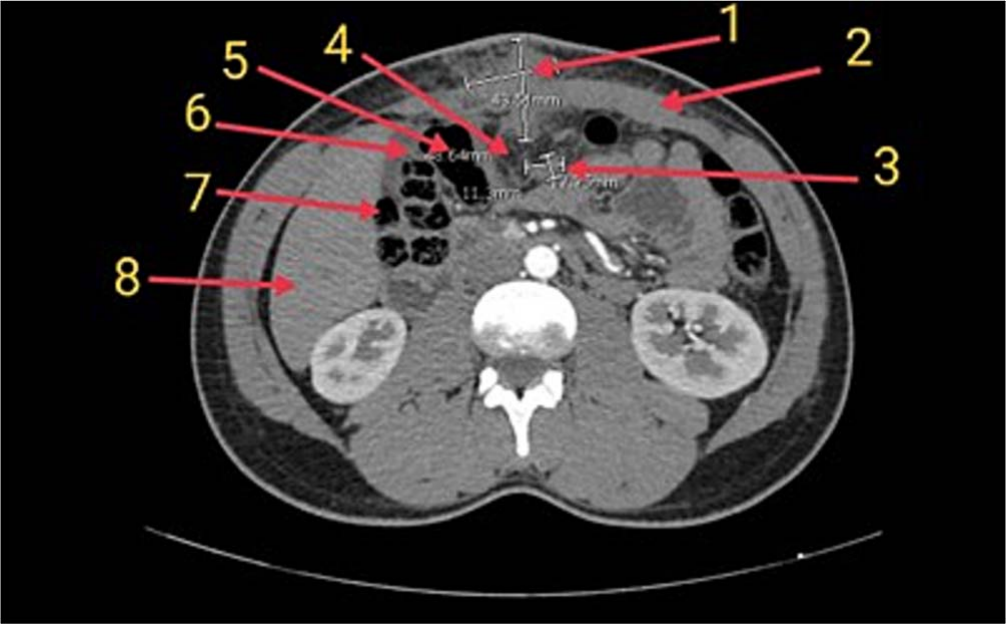
Axial CT image of the abdomen showing the inflammatory myofibroblastic tumor (1) in the supraumbilical anterior abdominal wall. The mass is closely associated with the rectus sheath (2), a presumed lymph node (3), and the greater omentum (4), which appears inseparable from the tumor. Adjacent bowel loops including the transverse colon (5), hepatic flexure (6), and ascending colon (7) are noted in close proximity. The liver is also partially visible (8) in the field.

Ultrasonography revealed a heterogeneous supraumbilical lesion extending intramuscularly and into the intra-abdominal cavity, measuring up to 6 cm, with surrounding edema and increased vascularity. MRI demonstrated an ill-defined, heterogeneously enhancing mass measuring approximately 5.2 × 5 cm, with low T1 and intermediate-to-high T2 signal intensity, associated fat stranding, and close proximity to adjacent bowel loops without a clear fat plane. Contrast-enhanced computed tomography (CT) confirmed extension through the rectus sheath with inseparability from the omentum and nearby colon, raising concern for an infiltrative process (Fig. 1).

Tru-Cut biopsy showed a spindle cell proliferation within fibrous and focally myxoid stroma, accompanied by mixed inflammatory infiltrates. Tumor cells demonstrated mild atypia and sparse mitotic activity. Immunohistochemistry was positive for smooth muscle actin, while CD34, ALK, S100, and β-catenin were negative, favoring a benign spindle cell tumor such as leiomyoma.

Given the lesion’s intra-abdominal extension and diagnostic uncertainty, surgical excision was performed. Through a supraumbilical incision, the mass was dissected from the anterior abdominal wall and separated from the transverse mesocolon. Complete excision with clear margins was achieved, and the abdominal wall was reinforced with mesh.

Gross examination revealed a firm yellow-gray mass measuring 4.5 × 2 × 1.5 cm within a larger musculo-fatty specimen. Histopathological analysis confirmed inflammatory myofibroblastic tumor, composed of myofibroblastic spindle cells with chronic inflammatory infiltrates, fibrosis, focal suppuration, and foamy histiocytes. No malignant features were identified, and all margins were tumor free. The postoperative course was uneventful, and the patient was scheduled for regular clinical and radiologic follow-up due to the risk of local recurrence.

## Discussion

Inflammatory myofibroblastic tumor is a rare mesenchymal neoplasm that predominantly affects children and young adults and may arise in both pulmonary and extrapulmonary locations [1, 4]. Involvement of the anterior abdominal wall is particularly uncommon, with only limited cases reported in the literature [5–7]. This rarity contributes to diagnostic uncertainty, especially when lesions present with nonspecific clinical and radiologic features.

The etiology of inflammatory myofibroblastic tumor remains unclear, although associations with inflammatory processes, trauma, and molecular alterations such as ALK gene rearrangements have been described [8]. ALK expression is more frequently observed in pediatric and pulmonary tumors, while extrapulmonary lesions in adults are often ALK negative, as in the present case [9].

Clinically, inflammatory myofibroblastic tumors typically present as slow-growing, painless masses without systemic symptoms [10]. Imaging findings are variable and may suggest aggressive behavior when tumors demonstrate ill-defined margins, infiltration, or close association with adjacent organs [9]. In our case, the proximity to bowel loops and loss of normal fat planes raised concern for malignancy [9].

Histopathological examination is essential for diagnosis, but core needle biopsy may be misleading due to limited tissue sampling and overlap with other spindle cell neoplasms such as leiomyoma, desmoid-type fibromatosis, or low-grade sarcoma [9]. This explains the initial misdiagnosis in the present case.

Complete surgical excision with negative margins remains the treatment of choice and is associated with favorable outcomes [11]. Although inflammatory myofibroblastic tumor is generally considered a tumor of intermediate biological potential, local recurrence and rare distant metastasis have been reported, particularly in ALK-negative tumors, warranting long-term follow-up [11].

In conclusion, complete surgical excision with negative margins remains the treatment of choice for inflammatory myofibroblastic tumor and is associated with favorable outcomes. Although generally considered a tumor of intermediate biological potential, inflammatory myofibroblastic tumor may recur locally and rarely metastasize, particularly in ALK-negative cases. Long-term clinical and radiologic follow-up is therefore recommended.
